# Determinants and Outcomes of Vasoplegia Following Left Ventricular Assist Device Implantation

**DOI:** 10.1161/JAHA.117.008377

**Published:** 2018-05-17

**Authors:** Kristen M. Tecson, Brian Lima, Andy Y. Lee, Fayez S. Raza, Grace Ching, Cheng‐Han Lee, Joost Felius, Ronald D. Baxter, Sasha Still, Justin D. G. Collier, Shelley A. Hall, Susan M. Joseph

**Affiliations:** ^1^ Baylor Heart and Vascular Institute Baylor Scott & White Research Institute Dallas TX; ^2^ Department of Internal Medicine Texas A&M University College of Medicine Health Science Center Dallas TX; ^3^ Department of Cardiovascular and Thoracic Surgery North Shore University Hospital Manhasset NY; ^4^ Department of Cardiology Baylor University Medical Center Dallas TX; ^5^ Annette C. and Harold C. Simmons Transplant Institute Baylor Scott & White Research Institute Dallas TX; ^6^ Department of Surgery Baylor University Medical Center Dallas TX; ^7^ Center for Advanced Heart and Lung Disease Baylor University Medical Center Dallas TX

**Keywords:** left ventricular assist device, vasoplegia, Cardiovascular Surgery, Heart Failure, Complications

## Abstract

**Background:**

Vasoplegia is associated with adverse outcomes following cardiac surgery; however, its impact following left ventricular assist device implantation is largely unexplored.

**Methods and Results:**

In 252 consecutive patients receiving a left ventricular assist device, vasoplegia was defined as the occurrence of normal cardiac function and index but with the need for intravenous vasopressors within 48 hours following surgery for >24 hours to maintain a mean arterial pressure >70 mm Hg. We further categorized vasoplegia as *none*;* mild*, requiring 1 vasopressor (vasopressin, norepinephrine, or high‐dose epinephrine [>5 μg/min]); or *moderate to severe*, requiring ≥2 vasopressors. Predictors of vasoplegia severity were determined using a cumulative logit (ordinal logistic regression) model, and 1‐year mortality was evaluated using competing‐risks survival analysis. In total, 67 (26.6%) patients developed mild vasoplegia and 57 (22.6%) developed moderate to severe vasoplegia. The multivariable model for vasoplegia severity utilized preoperative Interagency Registry for Mechanically Assisted Circulatory Support (INTERMACS) profile, central venous pressure, systolic blood pressure, and intraoperative cardiopulmonary bypass time, which yielded an area under the curve of 0.76. Although no significant differences were noted in stroke or pump thrombosis rates (*P*=0.87 and *P*=0.66, respectively), respiratory failure and major bleeding increased with vasoplegia severity (*P*<0.01). Those with moderate to severe vasoplegia had a significantly higher risk of mortality than those without vasoplegia (adjusted hazard ratio: 2.12; 95% confidence interval, 1.08–4.18; *P*=0.03).

**Conclusions:**

Vasoplegia is predictive of unfavorable outcomes, including mortality. Risk factors for future research include preoperative INTERMACS profile, central venous pressure, systolic blood pressure, and intraoperative cardiopulmonary bypass time.

**Clinical Perspective**

**What Is New?**

- In this first study to assess the severity of vasoplegia following left ventricular assist device implantation, we demonstrated that vasoplegia was a common complication, with 27% of patients developing a mild form and 23% developing a moderate to severe form, and that functional status, preoperative central venous pressure, preoperative systolic blood pressure, and cardiopulmonary bypass time were predictive of vasoplegia development and severity.

**What Are the Clinical Implications?**

- Because vasoplegia was associated with worse survival outcomes, future work is warranted to help inform physicians about how to prevent and/or mitigate vasoplegia.

## Introduction

Vasoplegia is a complication of surgery characterized by excessive vasodilation and low systemic vascular resistance that develops in 5% to 25% of patients undergoing cardiopulmonary bypass (CPB).^1^, ^2^ Although a number of studies have assessed vasoplegia in heart transplantation, data are sparse for advanced heart failure (HF) patients who receive a left ventricular assist device (LVAD). Preliminary studies have shown that HF patients are at risk of developing vasoplegia after LVAD surgery, but nothing has been reported, to our knowledge, to classify vasoplegia according to severity in LVAD recipients or to assess its effect on surgical outcomes. For these reasons, we sought to identify modifiable risk factors and to describe the consequences of vasoplegia following LVAD implantation.

## Methods

### Clinical

We compiled data on consecutive patients undergoing LVAD implantation surgery from June 2008 to May 2016 at the Baylor University Medical Center, after receiving approval from Baylor Scott and White Research Institute's institutional review board (with a waived requirement of informed consent). We followed the framework of Chan and colleagues by defining vasoplegia as the occurrence of normal cardiac function and index but with the need for intravenous vasopressors within 48 hours following surgery for >24 hours to maintain a mean arterial pressure >70 mm Hg.^3^ As in Esmailian and colleagues’ work, we further categorized vasoplegia as *none*;* mild*, requiring 1 vasopressor (vasopressin, norepinephrine, or high‐dose epinephrine [>5 μg/min]); or *moderate to severe*, requiring ≥2 vasopressors.^4^ The list of pre‐ and intraoperative variables examined are in Table 1. Postoperative outcomes were primarily evaluated using the Interagency Registry for Mechanically Assisted Circulatory Support (INTERMACS) definitions. A major bleed, for example, was defined as suspected internal or external bleeding resulting in death, reoperation, hospitalization, or transfusion of red blood cells (≥4 U packed red blood cells within any 24‐hour period during first 7 days after implantation). Furthermore, right HF (RHF) was defined as signs and symptoms of persistent right ventricular dysfunction following LVAD implantation and was categorized based on the duration of inotropes: *mild*, ≤7 days; *moderate*, 8–14 days; *severe*, >14 days; or *severe acute*, requiring a right ventricular assist device. Patients were followed for up to 1 year after transplant. The primary outcomes following transplantation were survival at 30 days and 1 year. The data, analytic methods, and study materials will not be made available to other researchers for purposes of reproducing the results or replicating the procedure.

**Table 1:** Preoperative and Intraoperative Sample Characteristics By Vasoplegia Severity

Variable	None (n=128)	Mild (n=67)	Moderate/Severe (n=57)	*P* Value
Preoperative				
Sex, male	104 (81.3)	51 (76.1)	48 (84.2)	0.81
Age, y	60 (49.5, 67.5)	58 (48, 67)	57 (49, 64)	0.47
Body mass index, kg/m^2^	28.7 (25, 33.9)	30.4 (23.9, 34.1)	30.6 (25.3, 35.6)	0.77
Diabetes mellitus	55 (43.0)	26 (38.8)	22 (38.6)	0.53
COPD	17 (13.3)	4 (6.0)	4 (7.0)	0.12
Destination therapy^10^	56 (44.8)	34 (52.3)	29 (55.8)	0.15
Prior sternotomy	46 (35.9)	21 (31.3)	18 (31.6)	0.50
Ischemic cardiomyopathy^1^	13 (10.2)	6 (9.0)	9 (15.8)	0.35
INTERMACS				<0.01
1	12 (9.4)	17 (25.4)	14 (24.6)	
2	43 (33.6)	19 (28.4)	28 (49.1)	
3	52 (40.6)	27 (40.3)	13 (22.8)	
4	21 (16.4)	4 (6.0)	2 (3.5)	
MELD score^1^	12 (10, 15)	14 (11, 16)	15 (12, 19)	<0.01
HeartMate II risk score^1^	1.7 (2.2, 1.2)	1.9 (1.4, 2.2)	2.1 (1.6, 2.6)	0.02
Medication (in past year)				
ACE inhibitor	71 (55.5)	39 (58.2)	30 (52.6)	0.81
Aldosterone	80 (62.5)	41 (61.2)	35 (61.4)	0.87
Amiodarone^1^	42 (33.1)	29 (43.3)	26 (45.6)	0.08
ARB^1^	15 (11.8)	4 (6.0)	11 (19.3)	0.29
Antiplatelet	86 (67.2)	39 (58.2)	42 (73.7)	0.62
β‐blocker	112 (87.5)	56 (83.6)	48 (84.2)	0.48
Warfarin^1^	53 (41.7)	21 (31.3)	18 (31.6)	0.13
Vasopressor‐dependent	12 (9.4)	17 (25.4)	14 (24.6)	<0.01
Heart rate, beats/min	84 (73.5, 95)	83 (77, 93)	95 (81, 107)	0.01
SBP, mm Hg^2^	109 (98, 121)	98 (90, 110)	99 (88.5, 111.5)	<0.01
DBP,^a^ mm Hg^2^	70 (62, 77)	64 (56, 73)	64.5 (60, 73)	0.04
MAP, mm Hg^2^	82 (75, 91)	74 (69, 85)	77 (70.5, 85)	<0.01
Systemic vascular resistance, PRU^10^	1288 (1021, 1643)	1261 (888, 1670)	1128 (909, 1450)	0.05
Serum creatinine, mg/dL	1.4 (1.1, 1.7)	1.4 (1.1, 1.9)	1.6 (1.3, 1.8)	0.03
LOS, d	4 (1, 9.5)	6 (2, 12)	7 (3, 13)	0.03
CVP, mm Hg^1^	13 (8, 17)	16 (12, 21)	18 (12, 22)	<0.01
Intraoperative				
Device type				0.67
HeartMate II	117 (91.4)	60 (89.6)	55 (96.5)	
HeartMate III	3 (2.3)	2 (3.0)	1 (1.8)	
HeartWare	8 (6.3)	5 (7.5)	1 (1.8)	
Concomitant procedure	31 (24.2)	24 (35.8)	22 (38.5)	0.03
CPB time, min^11^	69 (53, 85)	90.5 (72, 126)	92 (70, 119)	<0.01
Cross‐clamp use^3^	4 (3.1)	8 (11.9)	8 (14.0)	<0.01
Volume ultrafiltrated, mL^15^	1500 (0, 2725)	2000 (1200, 3900)	2000 (0, 3500)	0.03
Nadir hematocrit, %^2^	27 (24, 30)	26 (23, 30)	26 (24, 30)	0.89

Data are shown as frequency (%) or median (quartile 1, quartile 3). Superscripts indicate the number of missing values. Variables with *P*<0.05 were considered in a multivariable model (with the exception of MELD and HeartMate risk scores). ACE indicates angiotensin‐converting enzyme; ARB, angiotensin receptor blocker; COPD, chronic obstructive pulmonary disease; CPB, cardiopulmonary bypass; CVP, central venous pressure; DBP, diastolic blood pressure; INTERMACS, Interagency Registry for Mechanically Assisted Circulatory Support; LOS, length of stay; MAP, mean arterial pressure; MELD, Model for End‐Stage Liver Disease; PRU, peripheral resistance unit; SBP, systolic blood pressure.

a Diastolic blood pressure was normally distributed; the *P* value is from ANOVA.

### Statistical Analysis

We used the Wilcoxon rank sum test to examine differences in patient and surgical characteristics across vasoplegia severity for skewed continuous variables, and ANOVA was used for normally distributed variables. We used the Cochran–Armitage test for trend and the Kruskal–Wallis test to do the same for categorical variables. We also used the Cochran–Armitage test for trend to examine differences in postsurgical outcomes. Preoperative and intraoperative variables having significant relations with vasoplegia severity in bivariate analyses (ie, those with *P*<0.05 in Table 1) were considered jointly in a multivariable cumulative logit (ordinal logistic regression) model constructed via stepwise selection, using a significance level of 0.10 to stay in the model. Finally, we utilized the Fine and Gray method to assess the effect of vasoplegia severity on the outcome of survival while accounting for the competing risk of transplantation; we repeated the analysis after adjusting for age and INTERMACS profile.^5^ For simplicity, we refer to *survival* and *mortality* in this article, yet we truly mean *transplant‐free survival* and *transplant‐free mortality*, as we performed a competing‐risks analysis. Continuous variables are reported as median (quartile 1, quartile 3). Categorical variables are reported as frequencies and percentages. Analyses were performed using SAS version 9.4 (SAS Institute).

## Results

This analysis included 252 patients, of which 128 (50.8%) did not develop vasoplegia, 67 (26.6%) developed mild vasoplegia, and 57 (22.6%) developed moderate to severe vasoplegia following LVAD implantation. Overall, 203 (80.6%) patients were male, the median age was 59 (49, 66) years, and ≈92% (232) of devices implanted were HeartMate II (Thoratec). Eight patients (3.2%) used extracorporeal membrane oxygenation before LVAD placement; its use was not associated with vasoplegia severity (*P*=0.15). Generally, demographic information, comorbidities, and medication use were similar across vasoplegia severity categories (Table 1).

Among preoperative variables, we found that length of stay (LOS), serum creatinine, and central venous pressure (CVP) increased as the severity of vasoplegia worsened (*P*=0.03, 0.03, <0.01, respectively) (Table 1). The INTERMACS profile, MELD (Model for End‐Stage Liver Disease), and HeartMate II risk score all revealed a similar pattern; the worse the preoperative risk scores, the greater the vasoplegia severity. Those who required vasopressors before surgery were 3.2 times (95% confidence interval [CI], 1.6–6.6) more likely to develop a mild or worse form of vasoplegia following LVAD implantation (*P*<0.01). Higher values for preoperative systolic blood pressure (SBP), diastolic blood pressure, mean arterial pressure, and systemic vascular resistance exhibited protective effects against vasoplegia (*P*<0.01, *P*=0.04, *P*<0.01, and *P*=0.05, respectively). Those without vasoplegia, for example, had a median SBP of 109 mm Hg compared with a median of 99 mm Hg for those who had moderate to severe vasoplegia. Among intraoperative variables examined, CPB time, cross‐clamp use, and volume removed by ultrafiltration were all significantly higher as vasoplegia severity worsened (*P*<0.01, *P*<0.01, and *P*=0.03, respectively). Those who did not develop vasoplegia, for example, had a median CPB duration of 69 minutes, and those who developed moderate to severe vasoplegia had a median CPB duration of 92 minutes. No difference was observed in intraoperative nadir hematocrit.

**Figure 1:**
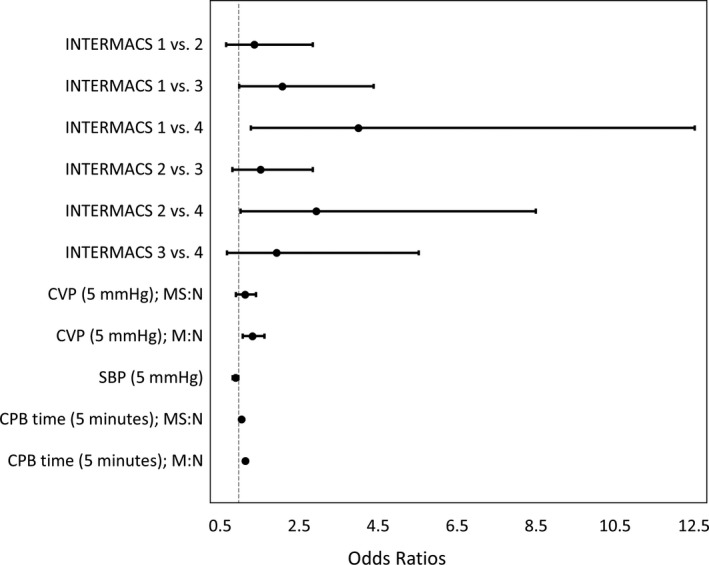
Odds ratios for the predictive classification model of vasoplegia severity. CPB indicates cardiopulmonary bypass; CVP, central venous pressure; INTERMACS, Interagency Registry for Mechanically Assisted Circulatory Support; M:N, odds ratio for the outcome of mild vasoplegia to no vasoplegia; MS:N, odds ratio for the outcome of moderate/severe vasoplegia to no vasoplegia; SBP, systolic blood pressure.

The multivariable model for vasoplegia severity utilized 4 variables: preoperative INTERMACS profile, CVP, SBP, and intraoperative CPB duration. This combination exhibited fair to good predictive ability with an area under the curve of 0.76. The model is fully described in Table 2, and the corresponding odds ratios are in Figure 1; note that this model utilized partial proportional odds. A patient with an INTERMACS profile of 2 is 2.94 times (95% CI, 1.02–8.50) more likely to develop a form of vasoplegia more severe than a patient with an INTERMACS profile of 4. In addition, for every 5 minutes of CPB, patients were 1.14 times (95% CI, 1.09–1.20) more likely to develop a mild or worse form of vasoplegia. Furthermore, an increase of 5 mm Hg of CVP increases the risk of developing worse severity of vasoplegia by 32% (odds ratio: 1.32; 95% CI, 1.07–1.62). In contrast, a 5‐mm Hg increase in SBP yields a 3% to 18% protective effect against any severity of vasoplegia (odds ratio: 0.89; 95% CI, 0.82–0.97).

**Table 2:** Maximum Likelihood Estimates for the Ordinal Logistic Regression Model for Vasoplegia Severity

Parameter	Vasoplegia	Estimate	SE	*P* Value
Intercept	Moderate/severe	−0.137	1.022	0.89
Intercept	Mild	−0.713	1.011	0.48
CVP	Moderate/severe	0.024	0.023	0.29
CVP	Mild	0.055	0.021	<0.01
CPB time	Moderate/severe	0.008	0.004	0.02
CPB time	Mild	0.026	0.005	<0.01
INTERMACS (1)	Any severity	0.606	0.273	0.03
INTERMACS (2)	Any severity	0.297	0.229	0.20
INTERMACS (3)	Any severity	−0.123	0.228	0.59
Systolic blood pressure	Any severity	−0.023	0.008	<0.01

Vasoplegia group *none* is the reference for intercept, CVP, and CPB time. CPB indicates cardiopulmonary bypass; CVP, central venous pressure; INTERMACS, Interagency Registry for Mechanically Assisted Circulatory Support.

Although no significant trend was noted in stroke rate or pump thrombosis, major bleeding events increased with vasoplegia severity (no vasoplegia: 7.8%; mild: 17.9%; moderate to severe: 31.6%; *P*<0.01; Table 3). Similarly, the rates of respiratory failure (ie, requiring mechanical ventilation following initial extubation) and LOS increased with vasoplegia severity. Furthermore, RHF also worsened with increased vasoplegia severity. None of the patients without vasoplegia had severe acute RHF compared with 11.9% of those with mild and 14.6% of those with moderate to severe vasoplegia (*P*<0.01). Alternatively, the rates of driveline infection decreased as vasoplegia worsened (none: 11.7%; mild, 4.5%; moderate to severe, 0.0%; *P*<0.01).

**Table 3:** Postoperative Sample Characteristics by Vasoplegia Severity

Variable	None (n=128)	Mild (n=67)	Moderate/Severe (n=57)	*P* Value
Major bleed	10 (7.8)	12 (17.9)	18 (31.6)	<0.01
RHF^a^				<0.01
None or mild	113 (91.1)	46 (68.7)	35 (63.6)	
Moderate	5 (4.0)	7 (10.5)	10 (18.2)	
Severe	6 (4.8)	6 (9.0)	2 (3.6)	
Severe acute	0 (0.0)	8 (11.9)	8 (14.6)	
Gastrointestinal bleed	17 (13.3)	6 (9.0)	5 (8.8)	0.31
Pump thrombosis	17 (13.3)	5 (7.5)	7 (12.3)	0.66
Driveline infection	15 (11.7)	3 (4.5)	0 (0.0)	<0.01
Respiratory failure	19 (14.8)	15 (22.4)	28 (49.1)	<0.01
Stroke	18 (14.1)	7 (10.5)	8 (14.0)	0.87
Postoperative LOS (d)	13.0 (10.0, 17.5)	16.0 (13.0, 24.0)	16.0 (14.0, 24.0)	<0.01
30‐d mortality^b^	1 (0.79)	6 (9.0)	10 (17.5)	<0.01
1‐y mortality^b^	24 (23.3)	16 (29.6)	18 (36.0)	0.10

Data are shown as frequency (%) or median (quartile 1, quartile 3). LOS indicates length of stay; RHF, right heart failure.

a Missing right heart failure status on 6 patients.

b Rates were calculated by removing transplanted patients from the denominator.

**Figure 2:**
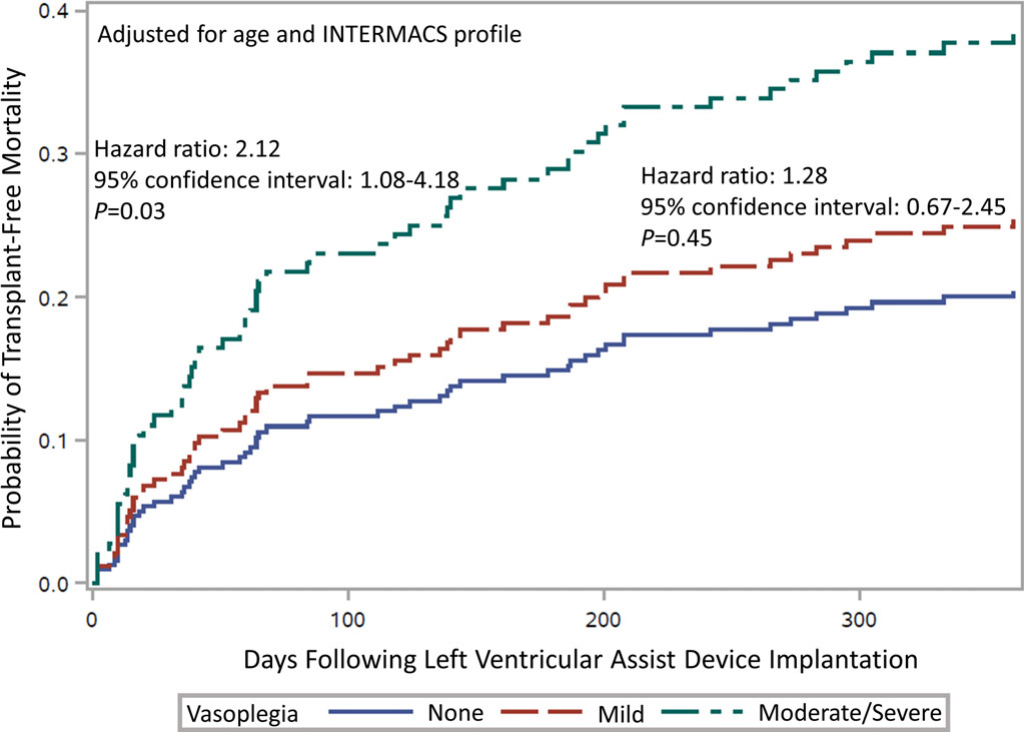
Cumulative incidence functions by severity of vasoplegia, adjusted by age and INTERMACS (Interagency Registry for Mechanically Assisted Circulatory Support) profile.

Within 30 days of LVAD implantation, only 1 patient (0.79%) without vasoplegia died, 6 (9.0%) with mild vasoplegia died, and 10 (17.5%) with moderate to severe vasoplegia died. The survival rates and curves differed significantly across the vasoplegia categories at 30 days (*P*<0.01). One‐year mortality rates increased across severity (23.3%, 29.6%, and 36.0%); however, the trend did not reach statistical significance (*P*=0.10). When considered as a time‐to‐event outcome, those with moderate to severe vasoplegia had a significantly higher risk of mortality than those without vasoplegia (adjusted hazard ratio: 2.12; 95% CI, 1.08–4.18; *P*=0.03). Alternatively, the risk of mortality for those with mild vasoplegia did not differ from those without vasoplegia (adjusted hazard ratio: 1.28; 95% CI, 0.67–2.45; *P*=0.45; Figure 2, Table 4).

**Table 4:** Hazard Ratios for Mortality (Competing Risks Analysis for Transplantation)

Model	Vasoplegia Category	Estimate	95% CI	*P* Value
Model 1	Mild vs none	1.38	0.74–2.58	0.32
	Moderate/severe vs none	1.99	1.08–3.69	0.03
Model 2	Mild vs none	1.28	0.67–2.45	0.45
	Moderate/severe vs none	2.12	1.08–4.18	0.03

Model 1: unadjusted. Model 2: adjusted for age and INTERMACS (Interagency Registry for Mechanically Assisted Circulatory Support) profile. CI indicates confidence interval.

## Discussion

Approximately half of the 252 patients in this analysis developed vasoplegia following LVAD implantation. We found that the best combination of variables to predict the severity of vasoplegia was preoperative INTERMACS profile, CVP, SBP, and intraoperative CPB duration. In addition, bivariate analyses revealed that preoperative serum creatinine and LOS as well as intraoperative cross‐clamp use and volume removed via ultrafiltration were associated with the development of vasoplegia. The severity of vasoplegia was associated with longer postoperative LOS, major bleeding, and mortality. In comparison to patients without vasoplegia, those with moderate to severe vasoplegia had more than double the risk of 1‐year mortality.

Rates of vasoplegia vary and depend heavily on the type of surgery and the clinical characteristics of the patient sample as well as the definition of vasoplegia. For example, vasoplegia defined as norepinephrine ≥0.2 μg/kg per minute for 12 to 24 hours in the intensive care unit was observed in 20% of HF patients undergoing elective cardiac surgery compared with 0% of similarly matched patients without HF undergoing the same surgery.^6^ A recent retrospective analysis reported a vasoplegia occurrence of 29%; however, only a small portion of that sample was comprised patients undergoing surgery for LVAD implantation (most had left ventricle restoration or CorCap [Acorn Cardiovascular] implantation).^7^ Furthermore, that study defined vasoplegia as the need for norepinephrine ≥0.2 μg/kg per minute and/or any dose of terlipressin combined with a cardiac index  ≥2.2 L/min per m^2^ for at least 12 consecutive hours within the first 3 postoperative days. In another study, ≈48% and 34% of patients undergoing CPB developed vasoplegia immediately and 24 hours following surgery, respectively, based on mean arterial pressure <50 mm Hg and the need for noradrenaline perfusion >0.08 μg/kg per minute.^8^ Another study reported a rate of 31% among heart transplant patients, with vasoplegia defined as “global systemic hypotension within 48 hours of heart transplantation with concurrent requirement of vasopressor administration (eg, vasopressin, epinephrine, or norepinephrine infusion of >5 μg/min) for >24 hours to maintain MAP [mean arterial pressure] >70 mm Hg.”^3^ Similarly, in the Esmailian heart transplant study, 34% developed vasoplegia (with the majority categorized as mild).^4^ We adopted Chan and colleagues’ definition of vasoplegia and observed a rate of 49%, which was the highest of the studies reviewed and may be explained by the greater severity of illness in LVAD patients compared with heart transplantation patients.

The use of CPB in cardiac surgery has been frequently linked with vasoplegia, with the rate of vasoplegia being higher for patients who have CPB than for those who have off‐pump coronary artery bypass surgery.^9^ Our study also reflected this inflammatory cascade, with worsening vasoplegia as CPB duration increased. The rates of concomitant procedures also increased across the vasoplegia severities, which may partly explain the increased CPB durations. In addition, both valvular surgery and surgery for the treatment of HF have been associated with the development of vasoplegia.^10^ Consequently, all participants in our study (having advanced HF and LVAD) were already susceptible to developing vasoplegia. Having a prior sternotomy has also been associated with the development of vasoplegia among heart transplant patients; however, this relation was not detected in our LVAD recipient cohort.^4^ Vasoplegia development has been directly associated with angiotensin‐converting enzyme inhibitors and heparin,^11^ whereas the use of β‐blockers exhibits an indirect relation (ie, a protective effect).^7^ We did not confirm the associations between those drugs and vasoplegia; however, patients in the previous studies were treated with angiotensin‐converting enzyme inhibitors and β‐blockers at much different rates than in our study. The use of amiodarone did trend toward significance in our study (*P*=0.08) and has been previously associated with vasoplegia in the heart transplantation population.^12^ In addition, the use of vasopressors before LVAD implantation has been associated with poor outcomes and RHF; we found that those with vasopressor dependence before surgery had >3 times the risk of developing vasoplegia.^13^ Our study revealed preoperative kidney dysfunction as being associated with vasoplegia, with those with moderate to severe vasoplegia having a median serum creatinine of 1.6 mg/dL compared with 1.3 mg/dL among those without vasoplegia. Also found to be predictive of vasoplegia severity in this study were CVP and SBP, which may be modified during the days of hospitalization before surgery. Relatedly, those who develop vasoplegia are less likely to have baseline hypertenstion.^7^, ^11^ Alternatively, another study failed to detect a difference in baseline CVP; however, it utilized patients with congenital heart defects and only 10 patients had vasoplegia, which raises concerns about statistical power and the appropriateness of generalization to the advanced HF population of interest.^14^

Although ours is the first model, to our knowledge, to predict vasoplegia severity, existing models are often used to allow surgeons to risk‐stratify patients before surgery. Although the MELD score was originally developed as a prognostic score for patients with end‐stage liver disease, it has proven helpful for risk prediction of mortality and RHF in patients undergoing heart transplantation and LVAD implantation.^15^, ^16^, ^17^ The HeartMate II risk score involves a combination of preoperative factors including age, albumin, serum creatinine, international normalized ratio, and center volume and is predictive of 90‐day mortality following LVAD implantation.^18^ That tool recently yielded controversial results of varying mortality rates across INTERMACS profiles, which suggested that the HeartMate II risk score may be superior to traditional INTERMACS profiling for risk stratification and that INTERMACS profiles alone should not disqualify a candidate from surgery.^19^ Our work demonstrated that the INTERMACS profile was more predictive of vasoplegia than the HeartMate II risk score.

The development of a reliable prediction tool is critical because vasoplegia has repeatedly been associated with poor surgical outcomes. Patients who developed vasoplegia following elective cardiac surgery, for example, had significantly worse 90‐day survival outcomes than those who did not develop vasoplegia (29% versus 9%).^7^ Another study reported a mortality rate of 50% for dialysis‐dependent patients who underwent CPB and subsequently developed vasoplegia.^9^ Our work reiterated the association between vasoplegia and mortality, as patients with moderate to severe vasoplegia had a >2‐fold increase in risk of mortality. In addition, we observed higher rates of major bleeding episodes among vasoplegic patients. This confirms the work of Chan and colleagues, who found a similar trend among transplant recipients, and Patarroyo with blood transfusions.^20^, ^21^ Although this finding may identify an area of confounding, the majority of bleeding events after LVAD implantation resolve within 24 hours. In our experience, 73% of the bleeding events resolved within 24 hours. Furthermore, patients developed respiratory failure at higher rates as vasoplegia severity worsened, and we found that those with moderate to severe vasoplegia developed RHF at ≈4 times the rate of those who did not have vasoplegia. As a result of these complications, the postoperative LOS for vasoplegic patients was significantly longer than for those without vasoplegia, a theme that has been reported previously.^22^ Contrary to all other surgical outcomes, vasoplegia was inversely related to driveline infection. This may be coincidental, or those with vasoplegia may have received prolonged antibiotics due to concerns about sepsis, which could have subsequently lowered the risk of driveline infection development.

Attempts have been made to prevent and mitigate the vasoplegia syndrome. In the United Kingdom, a liver transplantation department found that perfusing the liver at lower oxygen tensions yielded a lower occurrence of vasoplegia.^23^ One center advocated for implanting LVADs via minimally invasive procedures (ie, ministernotomy) to prevent vasoplegia.^24^ Similarly, our work indicates that reducing the time of CPB decreases the risk of developing vasoplegia. For patients who have already developed vasoplegia, a randomized clinical trial revealed that those who went on to receive vasopressin instead of norepinephrine had fewer postoperative complications.^25^ Furthermore, a literature review regarding the use of methylene blue (MB) as a treatment for vasoplegia confirmed that MB is safe if used within the recommended dosage and is useful for treating vasoplegia, although its benefit is time‐dependent.^26^ A newer study reiterated this finding; CPB patients who received MB in the operating room (early administration) had a mortality rate of 10.8% compared with 28.6% among those who received MB in the intensive care unit (late administration).^27^ Controversy exists regarding the use of MB in patients with both vasoplegia and right ventricular failure; however, it is generally accepted that its benefits outweigh the risks. Because Mehaffey and colleagues’ study was retrospective and observational, a significant knowledge gap remains regarding the potential usefulness of MB therapy; therefore, a prospective randomized trial may be warranted. We have not conducted formal research studies regarding MB; however, we have observed quick positive responses sustained for only a few hours.

### Limitations

This study has all the limitations of a small, retrospective review. These results were observed in a single center and may not be generalizable to other centers. At our center, for example, patients typically leave surgery on 1 high‐dose vasopressor, whereas other centers may use multiple low‐dose vasopressors. Because the data were not originally recorded with the intent to perform research, some variables were not available for analysis for all patients, such as echo parameters or a full listing of vasopressors and/or inotropes utilized. Similarly, patients’ medications are known only for the year before the operation and may not adequately reflect the medications used at the time of surgery. In addition, although we found CBP duration to be predictive of vasoplegia, it is possible that if severe hypotension develops while weaning from CPB, it may lead to somewhat longer CPB times until the hypotension is sufficiently controlled with vasopressors and/or other forms of support. Consequently, the strategy for coming off CPB includes utilizing moderate‐dose vasopressors/inotropes before separating from bypass. If blood pressure and cardiac output are adequate, then patients are weaned. If hypotension develops despite these preemptive pharmacologic maneuvers, it is often due to right heart dysfunction, which may need to be addressed via temporary right ventricular mechanical support.

## Conclusions

Our study of 252 LVAD recipients reaffirmed that the development of vasoplegia is a substantial risk following surgery and a predictor of unfavorable surgical outcomes, including death. Preoperative INTERMACS profile, CVP, SBP, and intraoperative CPB duration are all risk factors that may be assessed before and during LVAD implantation. Work is needed to determine whether modification of these risk factors, if possible, may limit the development and severity of vasoplegia following LVAD implantation surgery.

## Sources of Funding

This work was funded in part by the Baylor Health Care System Foundation.

## Disclosures

Drs Hall and Joseph have received speaking honoraria from Saint Jude Medical. The remaining authors have no disclosures to report.
